# Light-Induced Oxidase Activity of DNAzyme-Modified Quantum Dots

**DOI:** 10.3390/ijms21218190

**Published:** 2020-11-01

**Authors:** Krzysztof Żukowski, Joanna Kosman, Bernard Juskowiak

**Affiliations:** Department of Bioanalytical Chemistry, Faculty of Chemistry, Adam Mickiewicz University, Uniwersytetu Poznanskiego 8, 61-614 Poznan, Poland; krzysztof.zukowski@amu.edu.pl

**Keywords:** quantum dots (QDs), DNAzyme, ROS, Amplex Red, light-induced activity

## Abstract

Here, we report the synthesis of a quantum dot (QD)-DNA covalent conjugate to be used as an H_2_O_2_-free DNAzyme system with oxidase activity. Amino-coupling conjugation was carried out between amino-modified oligonucleotides (CatG4-NH_2_) and carboxylated quantum dots (CdTe@COOH QDs). The obtained products were characterized by spectroscopic methods (UV-Vis, fluorescence, circular dichroizm (CD), and IR) and the transmission electron microscopy (TEM) technique. A QD-DNA system with a low polydispersity and high stability in aqueous solutions was successfully obtained. The catalytic activity of the QD-DNA conjugate was examined with Amplex Red and ABTS (2,2′-azino-bis(3-ethylbenzothiazoline-6-sulfonate)) indicators using reactive oxygen species (ROS) generated by visible light irradiation. The synthesized QD-DNAzyme exhibited enhanced catalytic activity compared with the reference system (a mixture of QDs and DNAzyme). This proved the assumption that the covalent attachment of DNAzyme to the surface of QD resulted in a beneficial effect on its catalytic activity. The results proved that the QD-DNAzyme system can be used for generation of the signal by light irradiation. The light-induced oxidase activity of the conjugate was demonstrated, proving that the QD-DNAzyme system can be useful for the development of new cellular bioassays, e.g., for the determination of oxygen radical scavengers.

## 1. Introduction

Reactive oxygen species (ROS) are oxygen compounds that have a higher reactivity than molecular oxygen in the triplet state. The group of ROS mainly includes superoxide anion radicals (^•^O^2−^, ^•^O_2_^2−^), hydroxyl radicals (^•^OH), hydroperoxide radicals (HO_2_^•^), and oxygen species without an unpaired electron, such as singlet oxygen (^1^O_2_), ozone (O_3_), and hydrogen peroxide (H_2_O_2_) [1,2,3]. ROS are involved in many cellular processes. These include oxidative-phosphorylation coupling that occurs in mitochondria and provides energy for cell apoptosis or programmed self-destruction of the cells [4,5,6]. There are many methods employed for obtaining ROS, including biological [7,8,9], chemical [10,11], and electrochemical [12,13] approaches and methods that use photochemical or photocatalytic reactions [14,15].

Nowadays, semiconductor nanoparticles (NPs), for example, ZnO NP [16,17,18], TiO_2_ NP [19,20,21], and quantum dots (QDs) [22,23,24], are used to obtain oxygen free radicals. QDs are semiconductor nanocrystals with sizes ranging from 1 to 10 nm. These inorganic fluorophores are distinguished by a high quantum efficiency, narrow emission bands, a long life time of fluorescence, stability, and resistance to photobleaching [22,23,24]. The emission wavelength is directly related to the size of these nanoparticles, which gives the possibility to design a nanomaterial with emission at a specific wavelength. QDs can be easily modified, which provides a wide range of applications in medicine and diagnostics [25,26,27]. Another of their advantages is their semiconductor properties, which provide a wide range of potential applications of this type of system in the photovoltaic industry [28]. Considering the unique semiconductor properties of QDs, they can be used as efficient reactive oxygen generators. The application of QDs as ROS generators was proposed by the Niemeyer group [29]. They examined CdS QDs functionalized with an adsorbed layer of horseradish peroxidase (HRP) as ROS generators. The free oxygen radicals generated upon light irradiation were involved in the HRP-catalyzed oxidation of an indicator. The Campos-Terán group [30] used similar systems and focused on studying the effect of immobilization of the QD-enzyme on silanized silica for the photocatalytic application of this nanomaterial.

It is unclear whether the switching enzymatic activity of HRP/QDs by light is a unique phenomenon of protein enzymes [28,29], or whether this approach can also be used to design catalysts that comprise DNAzymes with peroxidase activity. Peroxidase-mimicking DNAzymes are promising alternatives to the HRP commonly used in bioanalytics. DNAzymes are based on the G-quadruplex (G4) architecture of some nucleic acids [31]. The planar structure of the G-quartet (part of the G-4 structure) is able to interact with other planar molecules, such as hemin. With the increasing strength of this end-stacking interaction, the peroxidase activity of hemin, being a cofactor in the resulting DNAzyme, also increases [31,32,33]. The strength of this interaction mainly depends on the topology of the G-quadruplex, which makes it an important factor affecting the DNAzyme catalytic capacity. Generally, the peroxidase activity of DNAzyme is also sensitive to the concentration and type of cations present in solution, the oligonucleotide sequence, and oligonucleotide strand modification. Undoubtedly, DNA-based systems have advantages over protein enzymes, because they are characterized by cheap and easy synthesis and modification, as well as a good stability at a higher temperature (increasing the range of potential applications). Working with nucleic acids also allows for the development of new analytical strategies based on the hybridization phenomenon that occurs between complementary DNA strands or target-aptamer complex formation [33,34,35].

**Figure 1:**
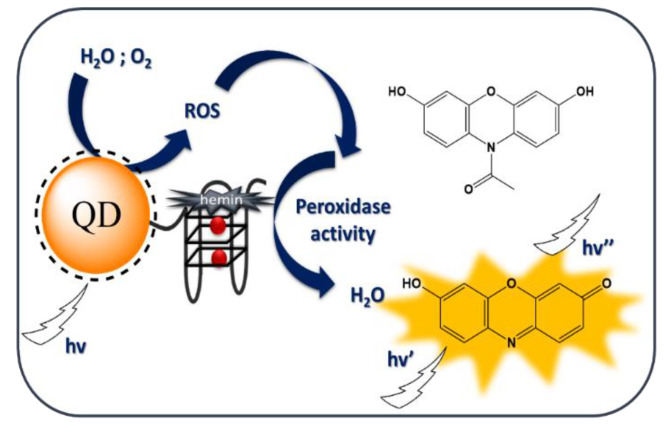
Expected mechanism of the oxidation reaction of the fluorogenic indicator Amplex Red catalyzed by the quantum dot (QD)-DNAzyme conjugate.

In this study, we examined the peroxidase activity of G-rich oligonucleotides attached to QDs. This system was designed as an oxidase-mimicking DNAzyme activated by light due to the QD generation of ROS (Figure 1). The DNAzyme-QD conjugate was characterized by spectroscopic methods (UV-Vis, fluorescence, circular dichroizm (CD), and IR) and transmission electron microscopy. The obtained QD-DNAzyme exhibited enhanced photocatalytic activity compared with the reference mixture of QD and DNAzyme. The activity of the QD-DNAzyme conjugate was mediated by light irradiation in the indicator reaction with Amplex Red.

## 2. Results and Discussion

### 2.1. Design and Synthesis of the QD-DNA Conjugate

**Figure 2:**
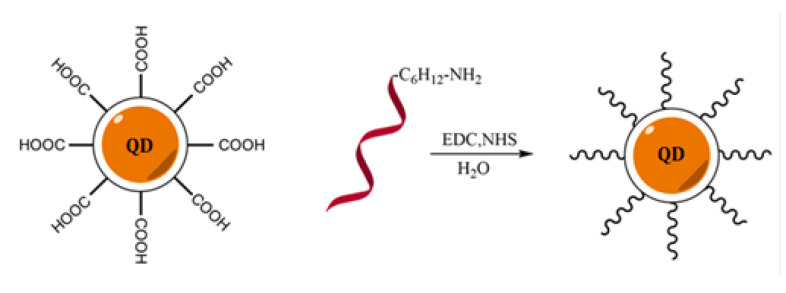
Scheme of the synthesis of the QD-DNA conjugate using amine coupling between COOH-functionalized quantum dots and amine-modified DNA oligonucleotides.

It has been proven that CdTe nanoparticles with a diameter of 2.7–3.0 nm possess a band gap of 2.4 eV and, upon visible light irradiation, are able to generate electrons which interact with oxygen and water molecules to form superoxide, hydroxyl radicals, singlet oxygen, and ROS [36]. It is also plausible that these forms of reactive oxygen are able to replace hydrogen peroxide in the oxidation reaction of organic substrates (for example, ABTS or Amplex Red) catalyzed by DNAzyme (scheme in Figure 1). In order to verify this hypothesis we designed a covalent QD-DNA conjugate to bring the DNAzyme closer to the surface of QD, where ROS are expected to be generated. The QD-DNA conjugate was obtained using an amine coupling reaction between carboxyl-functionalized QDs and amino-modified oligonucleotides. 1-Ethyl-3-(3-dimethylamino propyl) carbodiimide hydrochloride (EDC) and N-hydroxysuccinimide (NHS) were used as coupling agents (Figure 2) [37,38,39].

**Figure 3:**
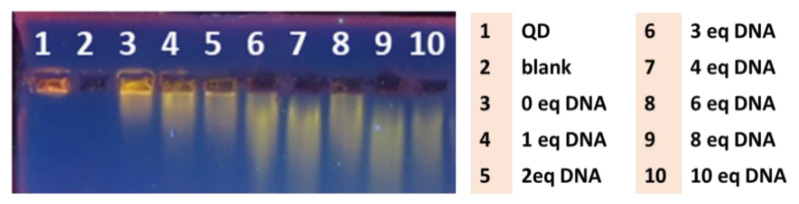
Agarose gel electrophoresis of QDs and QD-DNA conjugates (1% agarose gel, 1 × SB buffer (pH 8.5), 100 V). Stained with thiazole orange aqueous solution. Line 1—freshly prepared QDs alone; 2—DNA alone (blank); 3—QD after the reaction, no DNA was added; 4–10 products of the coupling reaction between QDs and varied concentrations of DNA.

In order to optimize the conditions of QD-DNA synthesis, the molar ratio of DNA:QD was investigated. The main goal was to obtain the conjugate product with the lowest polydispersity. A series of syntheses with different amounts of DNA equivalents were performed and analyzed using agarose gel electrophoresis (Figure 3). The mobility of the migrated QD band increased with the number of DNA equivalents used for QD-DNA synthesis. The mobility increase of the product can be explained by the successive attachment of anionic oligonucleotides to the nanoparticles that resulted in the more negative charge of the resulting conjugates. No further increase in the band mobility was observed for concentrations of DNA higher than 4 equivalents (Figure 3: lanes 7–10), which suggests saturation of the QD surface with oligonucleotides. The width of this relatively broad band was also unaffected at higher DNA/QD ratios, which proved completion of the coupling reaction with a relatively low polydispersity of the product. The sufficient amount of DNA used in the synthesis was 4–10 equivalents. Therefore, in the synthesis of QD-DNA for further studies, 8 equivalents of oligonucleotide over QDs were used to guarantee QD surface saturation.

In order to verify whether oligonucleotide molecules were covalently attached to the QD surface, the obtained QD-DNA conjugate was examined by a separation experiment with magnetic particles (Figure S1). Details of this experiment are described in the supplementary material. Briefly, the experiment was based on hybridization between an oligonucleotide (CatG4) covalently attached to QDs and a complementary strand (cCatG4) attached by a biotin–streptavidin interaction to a magnetic particle (Figure S1C). Visualization of the separated product under a UV light illuminator (Figure S1B) showed the bright luminescence of QDs, which proved that the covalent attachment of oligonucleotides to the QD surface was successful. 

**Figure 4:**
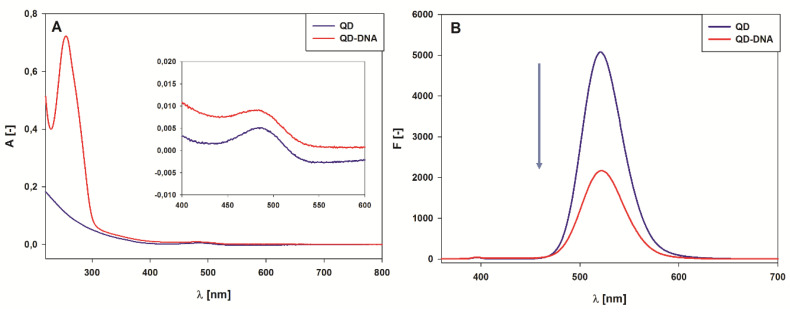
QD and QD-DNA spectral properties: UV-Vis absorption spectra (**A**) and emission spectra with excitation at 350 nm (**B**) in 10 mM Tris-HCl (pH = 8.0), and 1 µM QD and QD-DNA, respectively.

The information needed for further study of the QD-DNA conjugate was the average number of immobilized oligonucleotides on the surface of the nanoparticles. A direct estimation of the QD content in the QD-DNA conjugate using the fluorescence signal of QDs was hampered by the quenching effect of immobilized oligonucleotides (Figure 4B). Therefore, UV-Vis absorption spectroscopy was exploited for this purpose. The QD and oligonucleotide calibration graphs were plotted using absorption spectra recorded for series of standard solutions containing increasing concentrations of CdTe@COOH QDs or CatG4 oligonucleotides (Figure S2). Since QDs absorb at the same wavelength as CatG4 (Figure 4A), the QD absorption at 350 nm (beyond the DNA absorption band) was selected to quantify the QD concentration and to estimate the contribution of QD absorption to the total absorbance of QD-DNA conjugate at 260 nm. To determine the immobilization efficiency, expressed as a G4/QD molar ratio (number of CatG4 molecules immobilized on a single QD nanoparticle), a simple relationship (1) was used:

(1) $$G4/QD=\frac{A_{260}\varepsilon_{350}^{QD}}{A_{350}\varepsilon_{260}^{G4}}-\frac{\varepsilon_{260}^{QD}}{\varepsilon_{260}^{G4}},$$

where *A*_260_ and *A*_350_ are the absorbance for the *QD*-*DNA* conjugate, and ε parameters denote molar extinction coefficients for quantum dots (*QD* superscript) and CatG4 (*G4* superscript) at 260 or 350 nm (subscripts). The values of extinction coefficients ($\varepsilon_{350}^{QD}$ = 7.5 × 10^4^ M^−1^ cm^−1^, $\varepsilon_{260}^{QD}$ = 2.8 × 10^5^ M^−1^ cm^−1^, and $\varepsilon_{260}^{G4}$ = 1.8 × 10^5^ M^−1^ cm^−1^) were determined from calibration graphs (Figure S2). The calculated *G4*/*QD* ratio of 3.15 ± 0.3 indicates that, on average, three CatG4 molecules are immobilized on a single *QD* nanoparticle. This result is reasonable if one considers the surface area of about 25 nm^2^ for a single *QD*. The resulting area of 8 nm^2^ accessible for a 21-mer oligonucleotide seems to be reasonable for the extended single-stranded DNA adsorbed on the *QD* surface.

**Figure 5:**
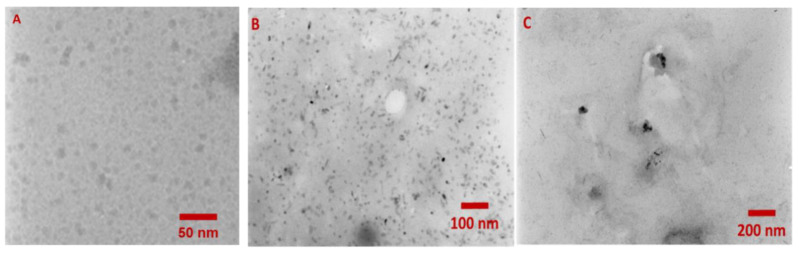
Transmission electron microscopy (TEM) images of nanoparticles: QDs (**A**); QD-DNA (**B**); QD/DNA mixture at a 1:3 molar ratio (**C**).

To estimate the size of the nanoparticles and their degree of polydispersity, the obtained products were characterized using transmission electron microscopy (TEM), dynamic light scattering (DLS), and zeta potential measurement (Table 1, Figure 5, Figures S3 and S4, Table S1). The zeta potential values for investigated nanoparticles are shown in Table 1. Three QD-containing systems were compared: Starting CdTe@COOH QDs; the QD-DNA conjugate; and the QD/DNA mixture at a 1:3 molar ratio that corresponded to the conjugate composition. The high negative value of the zeta potential (Table 1) proves that the covalent attachment of oligonucleotide molecules to the surface of the QDs has a beneficial effect on the stability of nanoparticles in aqueous solution. Transmission electron microscopy (TEM) was applied to characterize the size and polydispersity of the synthesized QD-DNA conjugate (Figure 5A–C). Unmodified CdTe@COOH nanoparticles displayed a dispersed form, as expected for nanoparticles with a surface covered with negatively charged carboxylic groups, which prevent aggregation (Figure 5A). Similar single nanoparticles could be observed for the DNA-modified QDs (Figure 5B). One can conclude that the obtained covalent QD-DNA conjugates are characterized by a high monodispersity (Figure 5B), in contrast to the QDs/DNA mixture (1:3), for which the formation of numerous small aggregates could be observed (Figure 5C). This aggregation may be explained by the more hydrophobic surface of QDs covered by physically adsorbed DNA molecules compared to the QD-DNA covalent conjugates. Size distribution histograms for QDs and the QD-DNA conjugate (Figure S3) indicated that nanoparticles after DNA conjugation retained their original size distribution. Unfortunately, the DLS results concerning the hydrodynamic size of the investigated systems disagreed with the TEM results (Figure S4, Table S1). The high values of hydrodynamic sizes obtained for all investigated systems can be explained by the aggregation of NPs in aqueous solution and possible fluorescence from QDs that contributed to the scattered light. Interestingly, the QD/DNA system exhibited larger aggregates than QDs and QD-DNA conjugates, in good agreement with the TEM results.

### 2.2. Spectroscopic Characterization of the QD-DNA Conjugate

The spectral properties of the QD-DNA conjugate and related systems were studied using UV-Vis absorption spectrophotometry, the fluorescence, and circular dichroizm (CD) spectroscopy. The UV-Vis spectrum of QDs displayed broad absorption, extending from the ultraviolet down to the band edge around 350 nm and a low intensity absorption band at 485 nm (Figure 4A and inset, blue line). In the case of the spectrum of QD-DNA (red line in Figure 4A), an additional absorption band emerged with a maximum at 260 nm, which should be ascribed to the absorption of oligonucleotide molecules immobilized on the surface of nanoparticles. The UV-Vis spectra proved that DNA oligonucleotides were immobilized on the surface of QDs.

As can be seen in Figure 4B, the emission spectrum of QD appeared to be sensitive to the modification of QDs with oligonucleotides. A significant decrease in the fluorescence intensity was observed for the QD-DNA system compared with unmodified QDs. The reference fluorescence spectrum for the QD/DNA mixture (non-covalent system, 1:3 ratio) also showed a quenching effect of oligonucleotides on the QD fluorescence, but one that was less efficient than for the QD-DNA covalent conjugate.

Fluorescence quenching titration of QDs was performed for three structural forms of CatG4: G-quadruplex (in the presence of K+); random coil conformation (without stabilizing ions); and with dsDNA (CatG4/cCatG4 hybrid). The high values of the Stern–Volmer constants suggested static quenching (Figure S5). All three forms of CatG4 have similar fluorescent quenching abilities (random coiled: K_SV_ = 6.0 × 10^4^ M^−1^, G-quadruplex: K_SV_ = 4.5 × 10^4^ M^−1^, and duplex: K_SV_ = 8.9 × 10^4^ M^−1^). The double-stranded form of DNA is able to quench fluorescence almost two times more efficiently than single-stranded oligonucleotides, but one should consider that dsDNA possesses two times more nucleobases than G4 and ssDNA forms of CatG4. Therefore, we assumed that this quenching was caused by the physical adsorption of DNA nucleobases on the surface of QDs, followed by a photoinduced electron transfer between QDs and nucleobases. Both forms of G-quadruplex and random coiled oligonucleotides quenched QD fluorescence, with a subtle difference showing that the G-quadruplex structure quenched the fluorescence less effectively. This difference also confirms that the process of the attachment of oligonucleotides to QDs was carried out successfully. The Zeng research group [40] noticed a similar effect for carboxyl-modified CdSe/ZnS 525QDs, which were quenched after the covalent conjugation of amino-modified oligonucleotides.

**Figure 6:**
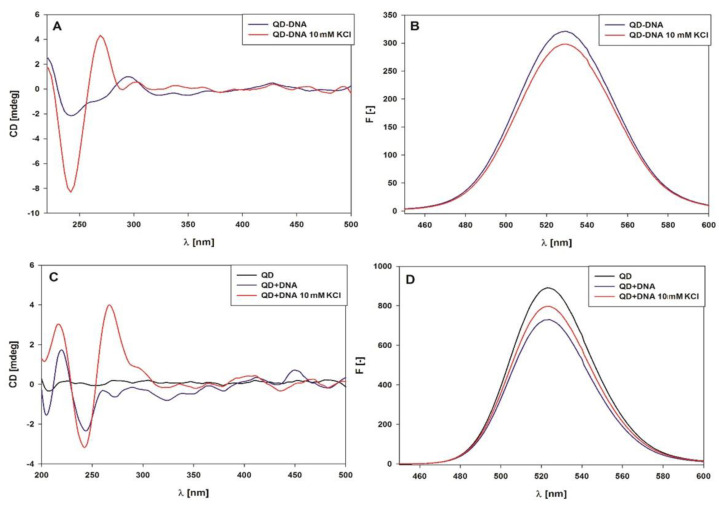
Circular dichroizm (CD) and fluorescence spectra of 1 µM QD-DNA (**A**,**B**) before (blue line) and after the addition of KCl at a 10 mM concentration (red line) and the spectra of 1 µM QD (**C**,**D**) before (black line) and after the addition of DNA at a 1:3 molar ratio (blue line) and the addition of KCl at a 10 mM concentration (red line).

It was also important to clarify whether the oligonucleotides on the QDs were able to form G-quadruplexes upon the addition of KCl. For this purpose, the CD spectra were analyzed. Figure 6A shows the CD spectra of the QD-DNA conjugate in the absence and presence of 10 mM KCl. This concentration of KCl is sufficient to transform unfolded CatG4 into a G-quadruplex structure [31,32,33,34]. Weak CD bands are present in the spectrum without KCl, which proves the lack of G-quadruplex assemblies on the QD surface (blue line). On the contrary, the CD spectrum in the presence of KCl (red line, Figure 6A) shows a strong negative band at 240 nm and positive band at 260 nm, proving the formation of a parallel G-quadruplex structure typical for the CatG4 sequence in the presence of potassium ions. For comparison, Figure 6C shows the CD spectra of QD alone and with added DNA (1:3 molar ratio, no KCl), as well as in the presence of 10 mM KCl. The band that appears at 265 nm after the addition of KCl also proves the formation of the G-quadruplex structure in this system. Another interesting observation was the increase in QD fluorescence when potassium ions were added to the system containing QD and DNA (Figure 6B,D). This effect agrees with the slightly lower K_SV_ value obtained for G4 DNA quenching (Figure S5). The random coil oligonucleotide can probably lay on QDs and more strongly interact with the QD surface than the G-quadruplex structure.

**Figure 7:**
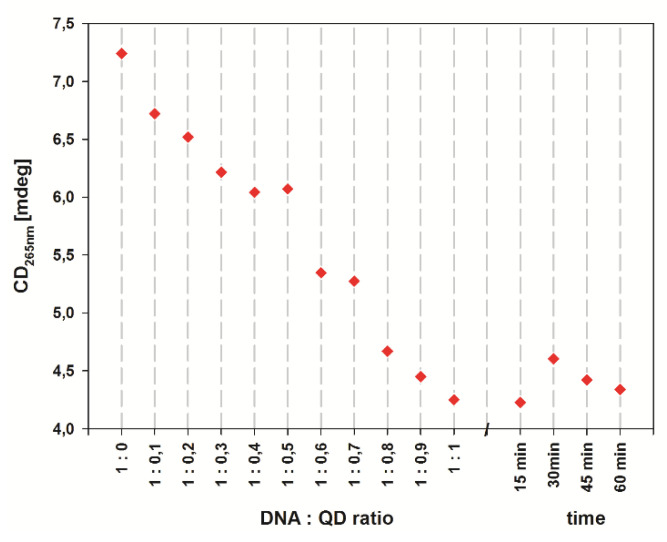
Scatter plot showing CD signal changes at 265 nm for 1 µM CatG4 in 10 mM KCl titrated with QDs. Additionally, after titration, the CD signal was subsequently recorded at intervals of 15 min.

The QD effect on the G-quadruplex structure stability was further investigated by titration of the G-quadruplex solution in 10 mM KCl with QDs (Figure S6). As shown in the titration plot (Figure 7), an increase in the QD concentration resulted in a moderate drop of the intensity of CD signals. This observation proved the modest destabilizing effect of QDs on the G-quadruplex structure of CatG4 oligonucleotides. Equilibrium in the system was reached immediately, since the CD spectra recorded within 1 h after the last QD addition revealed negligible spectral changes (Figure 7). The mechanism behind the decrease of CD signals probably involves competition between two processes: K^+^-stabilized G-quadruplex formation and the adsorption of an unstructured layer of oligonucleotides on the QD surface. The different behavior of the QD-DNA conjugate and QD/DNA system suggests the importance of the covalent immobilization of CatG4 for G-quadruplex structure formation on the QD surface.

### 2.3. Peroxidase Activity of the QD-DNA Conjugate

All peroxidase activity experiments were carried out for the systems that contained hemin at a 1:1 ratio to G4 DNA. The association of hemin changed the QD-DNA conjugate into a QD-DNAzyme catalyst. Two substrates for the catalytic reaction were studied: A colorimetric indicator called ABTS and a fluorogenic one known as Amplex Red. The reaction was preceded by sample irradiation with light (λ = 350 nm) for 5 min, in order to produce free oxygen radicals, which could then oxidize substrates in the DNAzyme catalyzed reaction (Figure 1). Initial experiments were carried out with the colorimetric substrate ABTS and typical experimental results are shown in Figure S8. Unexpectedly, a decrease in absorbance in the broad spectral range from 400 to 870 nm, including the absorption band at 414 nm, was observed. ABTS is known as a radical producing substrate and in an aqueous solution, a small fraction of the colored radical form ABTS^+●^ exists in equilibrium with the colorless ABTS molecules. Typically, in catalyzed reactions with hydrogen peroxide, an increase in absorption bands at 414 nm is observed as a result of the increase in the concentration of the ABTS^+●^ radical form. The observed decrease in the absorption band of the radical with catalytic reaction progress suggests that recombination and quenching processes occur between ROS produced by QD irradiation and the radicals formed by ABTS oxidation. As shown in Figure S8B, the QD-DNA conjugates caused less rapid bleaching in the ABTS^+●^ absorption bands compared with systems containing unmodified QDs (DNA/QD). Nemeyer et al. [29] reported similar results for the oxidation of ABTS catalyzed by HRP/CdS QDs. However, in their system, the oxidation of ABTS produced initially colorful products, followed by a decrease in absorbance at around 414 nm. They explained the observed effect by the further oxidation of ABTS^+●^ to the colorless ABTS^2+^ product. DNAzymes usually display lower activity than HRP. Therefore, we could not observe such an initial absorbance increase. Another reason for the lower activity of QD-DNAzyme compared with that for the QD/HRP system stems from the different method of QD irradiation, as well as the different type of QDs, used by Nemeyer et al. (CdS produces a higher number of ROS than CdTe QDs) [41].

**Figure 8:**
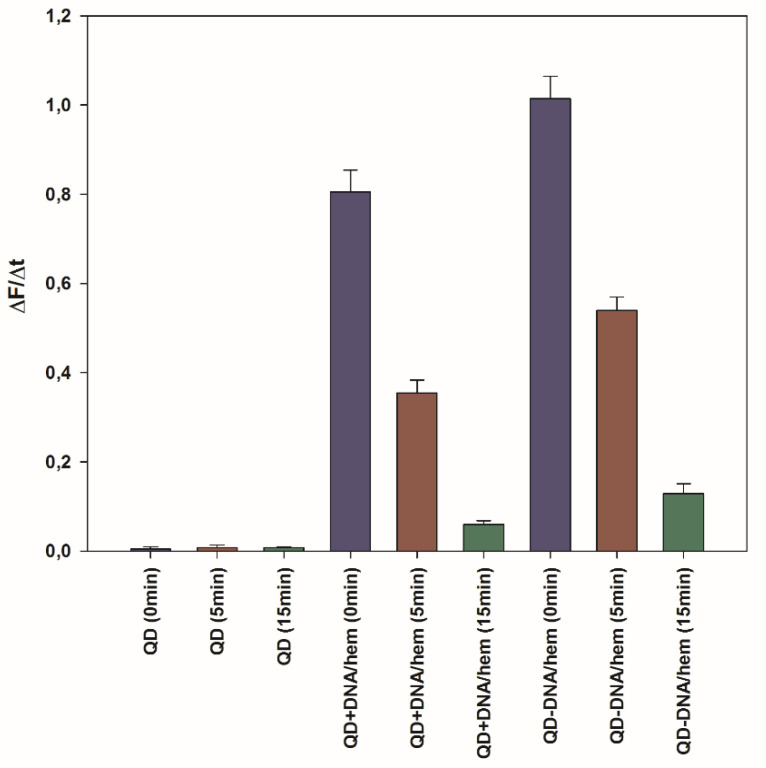
Relative oxidase activity for studied systems with Amplex Red substrate in 10 mM Tris-HCl (pH = 8.0). Conditions: 10 mM KCl; 33 nM QD-DNA or QDs; 100 nM DNA; 100 nM hemin; 5 µM Amplex Red; 1.1% DMSO. Samples were deaerated with Ar purging (0, 5, and 15 min) and irradiated with 350 nm light for 15 min prior to Amplex Red addition.

The activity of DNAzyme on the QD surface was then examined using the oxidation reaction of the fluorogenic substrate Amplex Red. Progression of the reaction can be easily followed, since the product of the reaction—resorufin—exhibits strong fluorescence. Moreover, contrary to ABTS^+●^, resorufin is not a radical product, so should remain stable in the experimental conditions. The initial rates of Amplex Red oxidation were determined from the first 5 min of the reaction and the results for different systems are shown in Figure 8.

**Figure 9:**
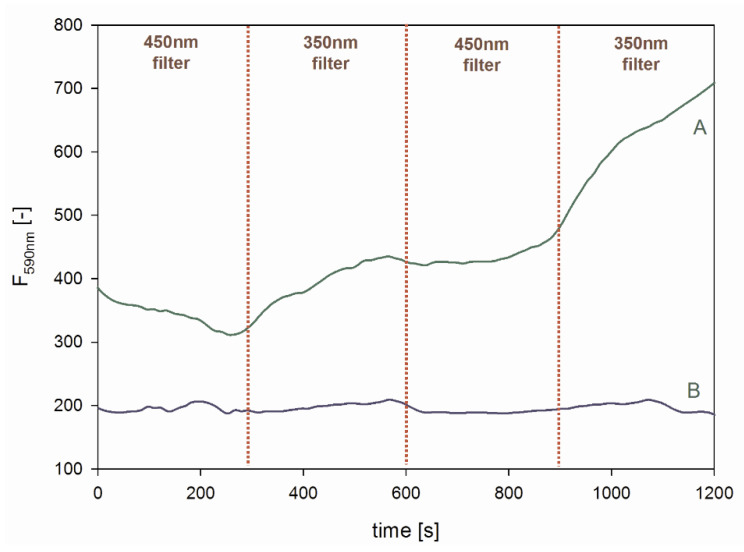
Light triggered the activity of QD-DNAzyme in the catalytic oxidation of Amplex Red (Trace (**A**)). Trace (**B**) represents the reference experiment without QD-DNAzyme. Fluorescence traces represent generation of the Amplex Red oxidation product (resorufin). The reaction system contained 33 nM QD-DNA, 100 nM hemin (100 nM DNAzyme), and 5 µM Amplex Red in 10 mM KCl.

As can be noticed, the QD-DNAzyme system exhibited higher activity towards the Amplex Red oxidation than the QD/DNAzyme mixture. It is also clear that there was no oxidation of the organic substrate in the reference systems without DNAzyme (buffer and QDs). In the case of deoxygenated solutions (5 or 15 min Ar purging and 15 min of irradiation by monochromatic light at 350 nm), the progression of resorufin production decreased dramatically for all investigated systems, in accordance with the reduced amount of generated ROS. This drop in catalytic activity was the most evident for extensively deoxygenated solutions (15 min Ar purging). The observed residual activity may be due to the production of hydroxyl radicals, together with ROS. The oxidation of Amplex Red was also performed for QD-DNAzyme systems with and without light irradiation, which produced ROS (Figure 9). In this experimental setup, the QD-DNAzyme/Amplex Red system was placed in the spectrofluorimeter and the sample was irradiated with light sequentially, through 350 and 450 nm long-pass filters at regular time intervals of 5 min. The fluorescence at 590 nm was monitored to follow the resorufin production. In both filter modes, resorufin could be excited, but only with irradiation through the 350 nm filter were QDs able to generate ROS. As can be seen in Figure 9 (trace A), the initial irradiation through a 450 nm filter did not enhance fluorescence, and even caused a fluorescence decrease. On the contrary, replacement of the 450 nm filter with the 350 nm one induced a noticeable increase in the fluorescence signal of resorufin. Subsequent replacement with the 450 nm filter again resulted in a horizontal run of the fluorescence signal, followed by a further increase in fluorescence after filter replacement. A reference experiment was carried out with Amplex Red in the absence of the QD-DNAzyme conjugate, which proved that the observed fluorescence changes were not caused by the photooxidation of Amplex Red (Figure 9, trace B). To confirm that the increase of recorded fluorescence was due to resorufin production, we recorded the fluorescence spectra of the system before and after 15 min irradiation through 450 and 350 nm filters, respectively. The spectra shown in Figure S9 confirmed that resorufin was the product of the catalytic reaction, which could be mediated by light. Undoubtedly, the short wavelength light (350 nm long-pass filter) induced the catalytic activity of DNAzyme due to the QD generation of ROS in the system.

These results prove that DNAzyme can catalyze the reaction between the indicator and ROS produced by QD light irradiation. The QD-DNAzyme system can be used for the development of assays for ROS scavengers (e.g., mannitol and sodium azide). This reaction system can also be exploited as a novel indicator reaction for DNAzyme-related study and used for the development of new biosensors and aptasensors for DNA and other analytes. We proved that the immobilization of DNA on QDs increases the activity of DNAzyme. Furthermore, this approach can be applied, for example, in the assessment of various QDs’ toxicity caused by the generation of ROS, especially in cellular conditions. The obtained results also present prospects for further research on covalent QD-DNA systems.

## 3. Materials and Methods

### 3.1. Materials

Studies were performed using CdTe@COOH QDs (Sigma Aldrich) and amino-modified oligonucleotide CatG4-NH2 (H2N-C6H12-5′-TGGGTAGGGCGGGTTGGGAAA-3′) purchased from Genomed (Poland). The oligonucleotide was HPLC-purified and its concentration was quantified by UV-Vis spectroscopy at 85 °C, with the following extinction coefficients at 260 nm: A = 15400; T = 8700; G = 11500; and C = 7400 (M*^−^*^1^ cm) [42]. Amplex Red (10-acetyl-3,7-dihydroxyphenoxazine), ABTS (2,2′-azino-bis(3-ethylbenzothiazoline-6-sulfonate)), 1-ethyl-3-(3-dimethylaminopropyl) carbodiimide hydrochloride (EDC), N-hydroxysuccinimide (NHS), and hemin were purchased from Sigma-Aldrich and used without any further purification. Hemin and Amplex Red were dissolved in DMSO and stored in a freezer (−30 °C).

### 3.2. Synthesis and Purification of QD-DNA Conjugates

QD-DNA conjugates were obtained using an amine coupling reaction between carboxylic group-coated QDs (CdTe@COOH) and amino-modified oligonucleotides based on the CatG4 sequence (H_2_N-C_6_H_12_-5′-TGG GTA GGG CGG GTT GGG AAA-3′). This reaction was promoted by EDC and NHS addition. The first step included 15 min incubation of modified QDs (1 equivalent; 2 nmol; 85.6 µL 23.4 µM) with freshly prepared NHS (200 equivalents; 400 nmol; 10 µL 40 mM) and EDC (400 equivalents; 800 nmol; 20 µL 40 mM) in 10 mM Tris-HCl buffer (pH = 8.0). The concentration of DNA was optimized and the experiments were performed using 1–10 equivalents of CatG4 oligonucleotide, with 8 equivalents being determined as an optimal ratio of DNA to QD. The final reaction with 8 equivalents of amino-modified oligonucleotide (16 nmol; 41.2 µL 388.3 µM) was carried out by incubation of the reaction mixture for 45 min on a magnetic stirrer (25 °C).The total volume of the reaction medium was 200 µL. The crude product of the amine coupling of DNA to the QDs was purified using 10 kDa cut-off centrifugal filter units (Amicon Ultra, Merck, Germany). For the experiments involving microscopic characterization and zeta potential measurements, the synthesis scale was 10 times higher.

### 3.3. Determination of the Size of Nanoparticles and their Zeta Potential

TEM images were collected using a JEM-1200EX transmission electron microscope (JEOL, Peabody, MA, USA). Prior to the visualization, the samples were applied to the copper/carbon TEM grids and allowed to evaporate. Measurements of the zeta potential and DLS experiments were conducted with a Nano ZS Zetasizer (Malvern, UK) equipped with an He-Ne laser of 633 nm using 23 µM aqueous solution of QDs alone, QD-DNA conjugate, and QDs with the addition of DNA at a 1:3 molar ratio. Parameters for the above experiments were material (CdTe) RI = 1.47, water RI = 1.330, and a viscosity of water equal to 0.8872 at 25 °C.

### 3.4. Spectroscopic Characterization of QD and QD-DNA Systems

QD and QD-DNA were characterized by UV-Vis, fluorescence, CD, and IR spectroscopy. Absorption spectra were recorded in the 220–800 nm range using a Jasco V-750 (Tokyo, Japan) spectrophotometer in 10 mm pathlength quartz cuvettes. Emission spectra were recorded with a Jasco FP-8200 spectrofluorimeter (Tokyo, Japan) in the range of 360 to 700 nm at a 350 nm excitation wavelength, using emission and excitation slits of 5 nm with a medium sensitivity of the detector. CD spectra were recorded using a Jasco J-1500 spectropolarimeter (Tokyo, Japan) in the spectral range from 220 to 500 nm at a rate of 200 nm/min and a number of accumulations equal to 3. The Jasco J-1500 spectropolarimeter, Jasco V-750 spectrophotometer, and FP-8200 spectrofluorimeter were equipped with Peltier temperature control accessories and all of the experiments were performed at 25 °C. IR spectra were recorded for aqueous solutions of QD and QD-DNA at 84 µM concentration using an ALPHA FT-IR spectrometer in a CaF_2_ cell.

### 3.5. Gel Electrophoresis

The electrophoresis experiment was performed in 1% agarose gel in 1 × SB buffer (pH = 8.50) for 45 min with a 100 V (10 V/cm) voltage using a B1A electrophoresis system model (Owl Separation Systems Llc., Portsmouth, NH, USA). After the electrophoresis process, gel was stained with thiazole orange and visualized using UV Transiluminator (Cleaver Scientific, Rugby, UK).

### 3.6. Activity Measurements

Measurements of DNAzyme activity against the generated ROS were carried out in 10 mm quartz cuvettes using the Jasco spectrofluorimeter, FP-8200 with a Peltier-type temperature accessory (irradiation and Amplex Red reaction measurements), and the Jasco V-750 spectrophotometer (ABTS reaction measurements). The samples containing QDs and other reagents were deoxygenated under argon for 0–15 min and then irradiated in a spectrofluorimeter at a wavelength of 350 nm with an excitation slit of 10 nm. A suitable indicator (fluorogenic—Amplex Red or colorimetric—ABTS) was added directly before the activity measurement started. Reactions with 5 µM Amplex Red indicator were carried out for samples containing 0.033 μM QD-DNA or QDs and 0.1 μM DNA for a reference QD/DNA system (the average ratio of DNA to QD of 3:1 was found in the QD-DNA conjugate). Reaction progression was monitored at λem = 590 nm with excitation at 560 nm. Experiments with ABTS indicator were carried out, with samples containing 0.167 μM QDs or QD-DNA, 0.5 μM DNA if needed, and 1 mM ABTS. Absorbance changes were monitored at 415 nm. In all cases, measurements were made in Tris-HCl buffer pH = 8.0 and 10 mM KCl, at a temp. of 25 °C. The concentration of hemin corresponded to the amount of G4-DNA at a 1:1 molar ratio.

### 3.7. Light-Induced Switching of Peroxidase Activity

In total, 33 nM QD-DNAzyme and 100 nM hemin in Tris-HCl buffer pH 8.0 were mixed in a quartz cell. Amplex Red stock solution was added to this such that the final concentration was 5 µM. The cell was placed in the spectrofluorimeter and the sample was irradiated with light sequentially through 350 and 450 nm long-pass filters at regular time intervals. The fluorescence at 590 nm was monitored to follow resorufin production. In both filter modes, resorufin was excited, but only with irradiation at the 350 nm filter did QDs generate ROS. A reference experiment was carried out with Amplex Red in the absence of the QD-DNAzyme conjugate.

## 4. Conclusions

The QD-DNA conjugate was successfully obtained by amino coupling, using EDC and NHS as a coupling reagents. The average number of immobilized oligonucleotide molecules on the surface of the nanoparticle was three, as proved by the spectroscopic method. It was also proven that the modification of QDs with DNA had a beneficial effect on the stability of the system in aqueous solutions. The obtained QD-DNA product was used as a DNAzyme system with oxidative activity triggered by light. This approach was based on the assumption that free oxygen radicals can be generated by QDs under the influence of light. The light-directed oxidative activity of the QD-DNAzyme conjugate was confirmed by oxidation of the fluorogenic Amplex Red indicator. There is a general lack of means for controlling and triggering the enzymatic activity of peroxidase-mimicking DNAzymes, for example, cellular applications are hampered because of the harsh reaction conditions due to the presence of H_2_O_2_, which makes the realization of in vivo applications of DNAzymes difficult. Taking into account the possibility to control the activity of the QD-based catalyst systems, we foresee applications of this light-switchable catalyst in biocatalysis, biosensing, and the design of novel cellular assays. The obtained results also present prospects for further research on covalent QD-DNAzyme systems (additional information Figure S7)

## Figures and Tables

**Table 1:** Values of the zeta potential measured using the dynamic light scattering method.

	Z (mV)	± ΔZ (mV)
QD	−41.9	3.85
QD + DNA	−55.0	1.44
QD − DNA	−47.1	4.69

## Supplementary Materials

The following are available online at https://www.mdpi.com/1422-0067/21/21/8190/s1, Figure S1: Visualization of magnetic particles experiment in Vis light (A) and under illumination with UV 254 nm (B). The reaction scheme of this experiment is shown in panel (C)., Figure S2: CdTe@COOH QD and CatG4-NH_2_ calibration plots for absorbance measured at 260 nm for DNA (A), QD (B) and at 350 nm for QD (C) in 10 mM Tris-HCl (pH = 8,0)., Figure S3: Size distribution obtain by TEM for QD (A) and QD-DNA conjugate (B)., Figure S4: Size distribution obtained with DLS technique for QD (A), QD-DNA (B) and QD+DNA 1:3 molar ratio (C). Zeta potential distribution for QD (D), QD-DNA (E), QD+DNA 1:3 molar ratio (F)., Figure S5: Stern-Volmer plots showing quenching of CdTe@COOH QDs fluorescence at 525 nm against DNA concentration. (A) G-quadruplex (CatG4 in 10 mM KCl), (B) ds DNA (CatG4/cCatG4 in 10 mM KCl), (C) ssDNA (CatG4 without KCl). 1 µM QDs in 10 mM Tris-HCl (pH = 8,0) was excited at 350 nm., Figure S6: Circular dichroism spectra of 1 µM CatG4 G-quadruplex (GQ) solution in 10 mM KCl titrated with QDs., Figure S7: Dependence of the fluorescence intensity of 1 μM QDs upon sequential titration with CatG4 and its complementary strand cCatG4., Figure S8: (A) Absorption spectra for QD-DNAzyme catalyzed oxidation of ABTS recorded at 0 - 1050 s, (B) Activity for studied systems with ABTS substrate in 10 mM Tris-HCl (pH = 8,0); 10 mM KCl; 167 nM QD-DNA or QD/500 nM DNA, 500 nM hemin; 1 mM ABTS, 0.1% DMSO. Activity was expressed by observing changes in absorbance at 415 nm with time (ΔA_415 nm_/Δt) and the irradiation time is 15 min using monochromatic light at 350 nm., Figure S9: Fluorescence spectra of resorufin produced by QD-DNAzyme catalyzed oxidation of Amplex Red, recorded before (black line), and after 15 min irradiation using 450 nm filter (red line) or 350 nm filter (green line). Emission spectra with excitation at 560 nm (Conditions: 10 mM Tris-HCl (pH = 8,0); 10 mM KCl; 33 nM QD-DNA (100 nM G-quadruplex), 100 nM hemin; 5 µM Amplex Red, 1,1% DMSO).

## References

[1] Halliwell B. (2006). Reactive Species and Antioxidants. Redox Biology Is a Fundamental Theme of Aerobic Life. Plant Physiol..

[2] Halliwell B., Gutteridge J.M.C. (1984). Oxygen Toxicity, Oxygen Radicals, Transition Metals and Disease. Biochem. J..

[3] Hayyan M., Hashim M.A., AlNashef I.M. (2016). Superoxide Ion: Generation and Chemical Implications. Chem. Rev..

[4] Kroemer G., Zamzami N., Susin S.A. (1997). Mitochondrial Control of Apoptosis. Immunol. Today.

[5] Marzo I. (1998). Bax and Adenine Nucleotide Translocator Cooperate in the Mitochondrial Control of Apoptosis. Science.

[6] Murphy M.P. (2009). How Mitochondria Produce Reactive Oxygen Species. Biochem. J..

[7] Liu H., Qu X., Kim E., Lei M., Dai K., Tan X., Xu M., Li J., Liu Y., Shi X. (2018). Bio-Inspired Redox-Cycling Antimicrobial Film for Sustained Generation of Reactive Oxygen Species. Biomaterials.

[8] Del Río L.A., López-Huertas E. (2016). ROS Generation in Peroxisomes and Its Role in Cell Signaling. Plant Cell Physiol..

[9] Cai P.-J., Xiao X., He Y.-R., Li W.-W., Zang G.-L., Sheng G.-P., Hon-Wah Lam M., Yu L., Yu H.-Q. (2013). Reactive Oxygen Species (ROS) Generated by Cyanobacteria Act as an Electron Acceptor in the Biocathode of a Bio-Electrochemical System. Biosens. Bioelectron..

[10] Walling C. (1975). Fenton’s Reagent Revisited. Acc. Chem. Res..

[11] Zepp R.G., Faust B.C., Hoign J. (1992). Hydroxyl Radical Formation in Aqueous Reactions (PH 3-8) of Iron(I1) with Hydrogen Peroxide: The Photo-Fenton Reaction. Environ. Sci. Technol..

[12] Katsounaros I., Cherevko S., Zeradjanin A.R., Mayrhofer K.J.J. (2014). Oxygen Electrochemistry as a Cornerstone for Sustainable Energy Conversion. Angew. Chem. Int. Ed..

[13] Katsounaros I., Schneider W.B., Meier J.C., Benedikt U., Biedermann P.U., Cuesta A., Auer A.A., Mayrhofer K.J.J. (2013). The Impact of Spectator Species on the Interaction of H2O2 with Platinum—Implications for the Oxygen Reduction Reaction Pathways. Phys. Chem. Chem. Phys..

[14] Surib N.A., Kuila A., Saravanan P., Sim L.C., Leong K.H. (2018). A Ligand Strategic Approach with Cu-MOF for Enhanced Solar Light Photocatalysis. New J. Chem..

[15] Liao Y., Brame J., Que W., Xiu Z., Xie H., Li Q., Fabian M., Alvarez P.J. (2013). Photocatalytic Generation of Multiple ROS Types Using Low-Temperature Crystallized Anodic TiO2 Nanotube Arrays. J. Hazard. Mater..

[16] Yin H., Casey P.S., McCall M.J., Fenech M. (2010). Effects of Surface Chemistry on Cytotoxicity, Genotoxicity, and the Generation of Reactive Oxygen Species Induced by ZnO Nanoparticles. Langmuir.

[17] Lipovsky A., Tzitrinovich Z., Friedmann H., Applerot G., Gedanken A., Lubart R. (2009). EPR Study of Visible Light-Induced ROS Generation by Nanoparticles of ZnO. J. Phys. Chem. C.

[18] Sirelkhatim A., Mahmud S., Seeni A., Kaus N.H.M., Ann L.C., Bakhori S.K.M., Hasan H., Mohamad D. (2015). Review on Zinc Oxide Nanoparticles: Antibacterial Activity and Toxicity Mechanism. Nano-Micro Lett..

[19] Brzicova T., Sikorova J., Milcova A., Vrbova K., Klema J., Pikal P., Lubovska Z., Philimonenko V., Franco F., Topinka J. (2019). Nano-TiO_2_ Stability in Medium and Size as Important Factors of Toxicity in Macrophage-like Cells. Toxicol. In Vitro.

[20] Chen L., Hu C., Guo Y., Shi Q., Zhou B. (2019). TiO2 Nanoparticles and BPA Are Combined to Impair the Development of Offspring Zebrafish after Parental Coexposure. Chemosphere.

[21] Aliakbari F., Haji Hosseinali S., KhaliliSarokhalil Z., Shahpasand K., Akbar Saboury A., Akhtari K., Falahati M. (2019). Reactive Oxygen Species Generated by Titanium Oxide Nanoparticles Stimulate the Hemoglobin Denaturation and Cytotoxicity against Human Lymphocyte Cell. J. Biomol. Struct. Dyn..

[22] Blaškovičová J., Sochr J., Koutsogiannis A., Diamantidou D., Kopel P., Adam V., Labuda J. (2018). Detection of ROS Generated by UV-C Irradiation of CdS Quantum Dots and Their Effect on Damage to Chromosomal and Plasmid DNA. Electroanalysis.

[23] Dou X., Zhang Q., Shah S.N.A., Khan M., Uchiyama K., Lin J.-M. (2019). MoS_2_ -Quantum Dot Triggered Reactive Oxygen Species Generation and Depletion: Responsible for Enhanced Chemiluminescence. Chem. Sci..

[24] Ribeiro D.S.M., Frigerio C., Santos J.L.M., Prior J.A.V. (2012). Photoactivation by Visible Light of CdTe Quantum Dots for Inline Generation of Reactive Oxygen Species in an Automated Multipumping Flow System. Anal. Chim. Acta.

[25] Chan W.C.W., Maxwell D.J., Gao X., Bailey R.E., Han M., Nie S. (2002). Luminescent Quantum Dots for Multiplexed Biological Detection and Imaging. Curr. Opin. Biotechnol..

[26] Biju V., Itoh T., Anas A., Sujith A., Ishikawa M. (2008). Semiconductor Quantum Dots and Metal Nanoparticles: Syntheses, Optical Properties, and Biological Applications. Anal. Bioanal. Chem..

[27] Jamieson T., Bakhshi R., Petrova D., Pocock R., Imani M., Seifalian A.M. (2007). Biological Applications of Quantum Dots. Biomaterials.

[28] Nozik A.J., Beard M.C., Luther J.M., Law M., Ellingson R.J., Johnson J.C. (2010). Semiconductor Quantum Dots and Quantum Dot Arrays and Applications of Multiple Exciton Generation to Third-Generation Photovoltaic Solar Cells. Chem. Rev..

[29] Fruk L., Rajendran V., Spengler M., Niemeyer C.M. (2007). Light-Induced Triggering of Peroxidase Activity Using Quantum Dots. ChemBioChem.

[30] Iñarritu I., Torres E., Topete A., Campos-Terán J. (2017). Immobilization Effects on the Photocatalytic Activity of CdS Quantum Dots-Horseradish Peroxidase Hybrid Nanomaterials. J. Colloid Interface Sci..

[31] Travascio P., Li Y., Sen D. (1998). DNA-Enhanced Peroxidase Activity of a DNA Aptamer-Hemin Complex. Chem. Biol..

[32] Kong D.-M., Yang W., Wu J., Li C.-X., Shen H.-X. (2010). Structure–Function Study of Peroxidase-like G-Quadruplex-Hemin Complexes. Analyst.

[33] Kosman J., Juskowiak B. (2011). Peroxidase-Mimicking DNAzymes for Biosensing Applications: A Review. Anal. Chim. Acta.

[34] Kosman J., Żukowski K., Juskowiak B. (2018). Comparison of Characteristics and DNAzyme Activity of G4–Hemin Conjugates Obtained via Two Hemin Attachment Methods. Molecules.

[35] Wang F., Lu C.-H., Willner I. (2014). From Cascaded Catalytic Nucleic Acids to Enzyme−DNA Nanostructures: Controlling Reactivity, Sensing, Logic Operations, and Assembly of Complex Structures. Chem. Rev..

[36] Goodman S.M., Levy M., Li F.-F., Ding Y., Courtney C.M., Chowdhury P.P., Erbse A., Chatterjee A., Nagpal P. (2018). Designing Superoxide-Generating Quantum Dots for Selective Light-Activated Nanotherapy. Front. Chem..

[37] Pong B.-K., Trout B.L., Lee J.-Y. Preparation of DNA-Functionalised CdSe/ZnS Quantum Dots. https://dspace.mit.edu/handle/1721.1/35870.

[38] Karakoti A.S., Shukla R., Shanker R., Singh S. (2015). Surface Functionalization of Quantum Dots for Biological Applications. Adv. Colloid Interface Sci..

[39] Lišková M., Voráčová I., Klepárník K., Hezinová V., Přikryl J., Foret F. (2011). Conjugation Reactions in the Preparations of Quantum Dot-Based Immunoluminescent Probes for Analysis of Proteins by Capillary Electrophoresis. Anal. Bioanal. Chem..

[40] Kubota Y., Motosa Y., Shigemune Y., Fujisaki Y. (1979). Fluorescence quenching of 10-methylacrjdinium chloride by nucleotides. Photochem. Photobiol..

[41] Torimura M., Kurata S., Yamada K., Yokomaku T., Kamagata Y., Kanagawa T., Kurane R. (2001). Fluorescence-Quenching Phenomenon by Photoinduced Electron Transfer between a Fluorescent Dye and a Nucleotide Base. Anal. Sci..

[42] Fasman G.D. (1975). Handbook of Biochemistry and Molecular Biology, Volume 1: Nucleic Acids.

