# Supplementary material for: Light-Induced Oxidase Activity of DNAzyme-Modified Quantum Dots
Source: Int J Mol Sci. 2020 Nov 1;21(21):8190. doi: 10.3390/ijms21218190 (PMC7662946; doi:10.3390/ijms21218190)
Supplement: Supplementary file 1 [file ijms-21-08190-s001.pdf]

International Journal of
Molecular Sciences

MDPI

Light-Induced Oxidase Activity of DNAzyme-Modified Quantum Dots

Krzysztof Żukowski, Joanna Kosman * and Bernard Juskowiak *

Supplementary Materials

1. Magnetic particles experiment – separation of QD-DNA conjugate

The streptavidin magnetic particles (SMPs) were first washed three times with TEN$_{100}$ buffer pH=7,4 (10mM Tris-HCl; 1mM EDTA; 100mM NaCl). Then the biotinylated oligonucleotide cCatG4-biotin (Biotin-5′-$T_8C_3A_2C_3GC_3TAC_3A$-3′) from Genomed (Poland) was attached to the streptavidin-modified magnetic particles for 1 h at room temperature upon stirring in TEN$_{1000}$ buffer (10mM Tris-HCl; 1mM EDTA; 1 M NaCl; pH=7.4). Next, after separation step with a neodymium magnet, the filtrate was discarded and the residue was washed three times with TEN$_{1000}$ buffer. After the last washing, 0.5 ml QD-DNA solution (10mM Tris-HCl (pH=8,0) with 100mM NaCl) was added. Whole solution was heated to 90^{0}C for 5 min and cooled slowly to room temperature for 3 hours. Then, the supernatant was discarded and the residue was washed with 10mM Tris-HCl (pH=8,0) buffer. In the next step, 0,5 mL of 10 mM Tris-HCl (pH=8,0) with 100 mM NaCl was added and the solution was mixed again. After that a neodymium magnet was attached to the Eppendorf wall to transport magnetic nanoparticles to the edge of the Eppendorf tube. Three independent samples were photographed in the visible light and under a UV illuminator.

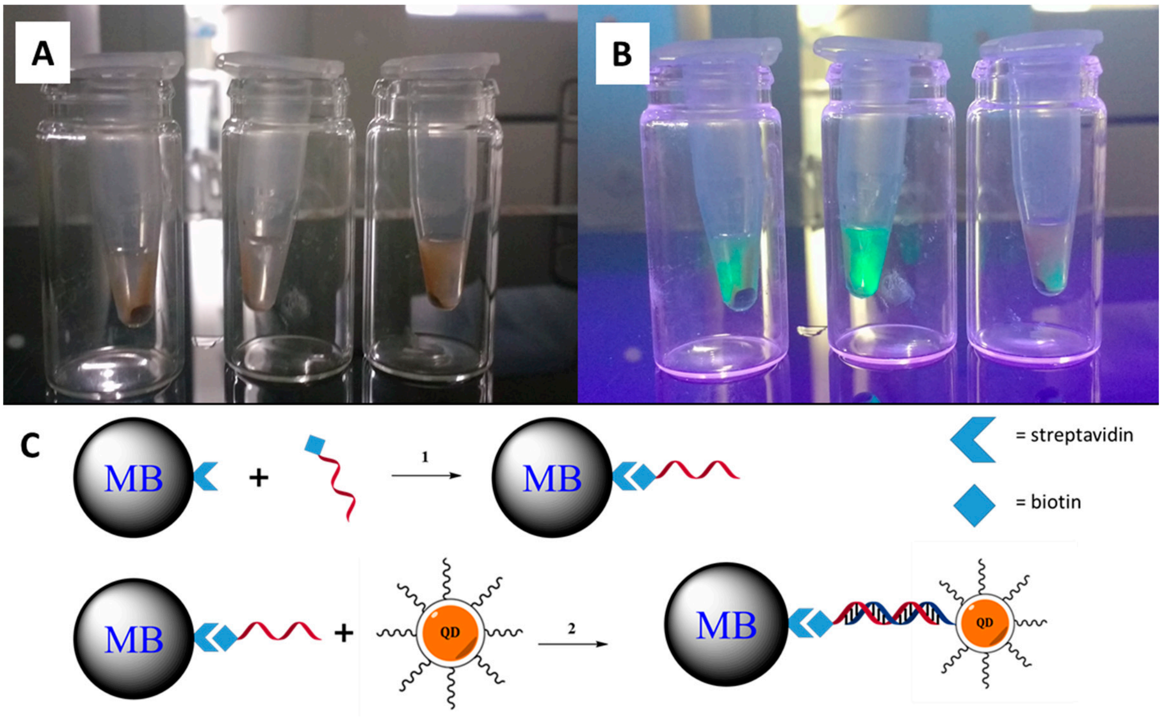

Figure S1. Visualization of magnetic particles experiment in Vis light (A) and under illumination with UV 254 nm (B). The reaction scheme of this experiment is shown in panel (C).

2. Determination of DNA/QD ratio and molar coefficients of DNA and QD

The molar extinction coefficients were determined from calibration graphs shown in Fig. S2. The following ε values were determined: $\varepsilon_{QD(260nm)}$ = (2.8±0.4x10^5; $\varepsilon_{DNA(260nm)}$ = (1.8±0.2)x10^5; $\varepsilon_{QD(350nm)}$ = (7.5±0.3) x10^4; [dm^{3}mol^{-1}cm^{-1}]. Concentrations of DNA and GQ in GQ-DNA conjugate and then the

DNA/QD ratio were calculated from separate equations: $C_{QD}=A_{350nm}/\varepsilon_{QD(350nm)}$ [M]; $C_{DNA}=(A_{260nm}-\varepsilon_{QD(260nm)}\cdot C_{QD})/\varepsilon_{DNA(260nm)}$ [M] or from the relationship (1) derived from these two equations.

$$G4/QD = \frac{A_{260}\varepsilon_{350}^{QD}}{A_{350}\varepsilon_{260}^{G4}} - \frac{\varepsilon_{260}^{QD}}{\varepsilon_{260}^{G4}} \quad (1)$$

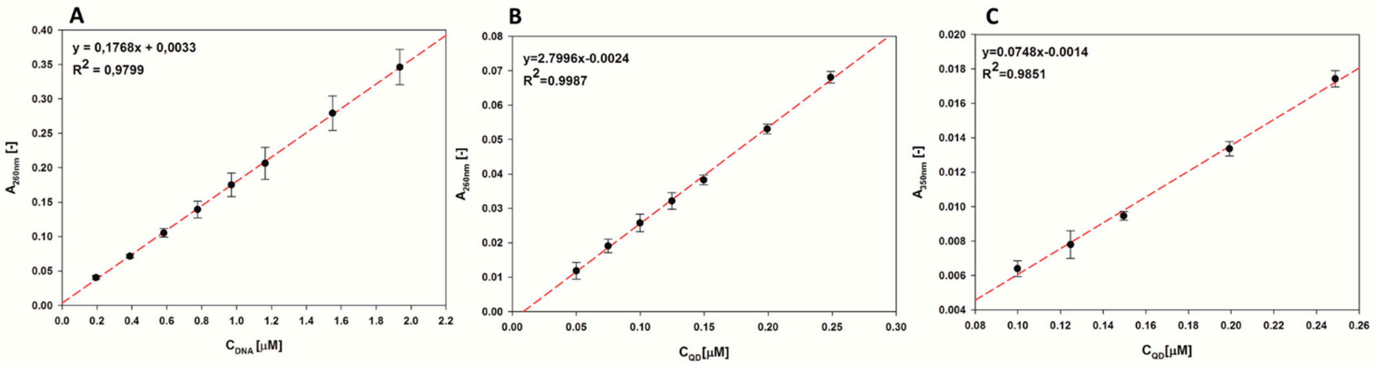

Figure S2. CdTe@COOH QD and CatG4-NH_2 calibration plots for absorbance measured at 260 nm for DNA (A), QD (B) and at 350 nm for QD (C) in 10 mM Tris-HCl (pH=8,0).

3. TEM, DLS and Zeta potential

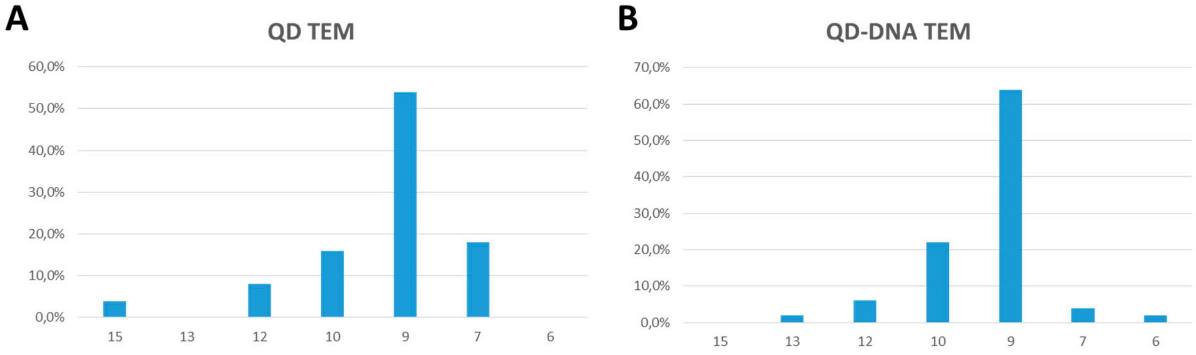

Figure S3. Size distribution obtain by TEM for QD (A) and QD-DNA conjugate (B).

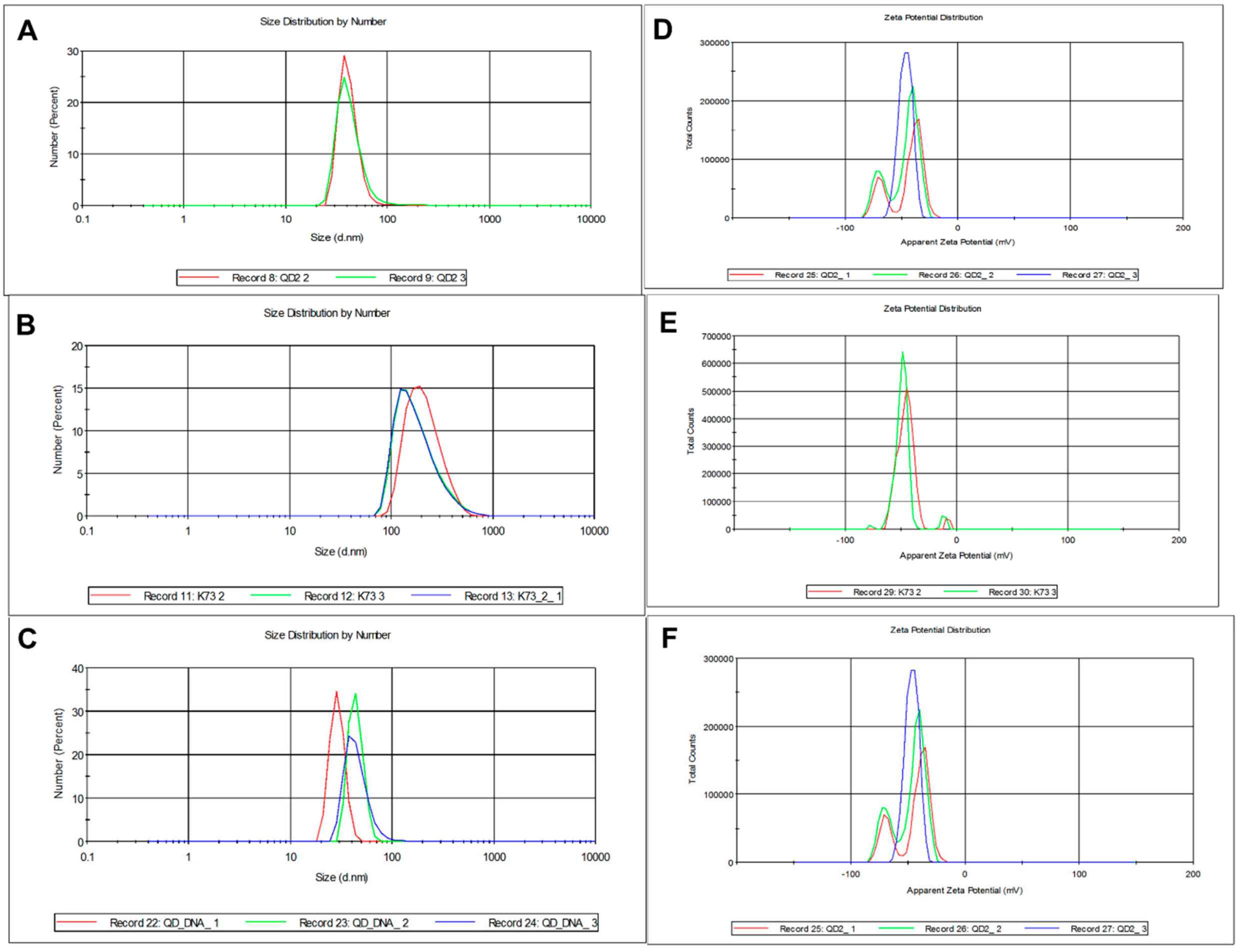

Figure 4. Size distribution obtained with DLS technique for QD (A), QD-DNA (B) and QD+DNA 1:3 molar ratio (C). Zeta potential distribution for QD (D), QD-DNA (E), QD+DNA 1:3 molar ratio (F).

Table 1. Comparison of size for QDs systems obtained with TEM and DLS techniques.

	Size from TEM [nm]	Size from DLS [nm]	PdI
**QD**	9.1 ± 1.6	42.8 ± 0.7 (>98%)	0.4405 ± 0.0285
**QD-DNA**	9.3 ± 1.2	39.5 ± 7.4 (>98%)	0.3607 ± 0.0103
**QD+DNA (1:3)**	-	197.3 ± 13.5 (>98%)	0.6687 ± 0.0365

3. Fluorescence titration of QDs with CatG4 oligonucleotide

These measurements were carried out by titrating an aqueous solution of quantum dots (1 μM) with small amounts (5 μL) of aqueous solution of G4-DNA/dsDNA (100 μM). After each addition of DNA and incubation for 5 min, emission spectra were recorded with 350 nm excitation, ex/em slits of 5nm, medium sensibility of PMT using a Jasco FP-8200 (Japan) spectrofluorimeter at 25°C and 10 mm quartz cuvettes.

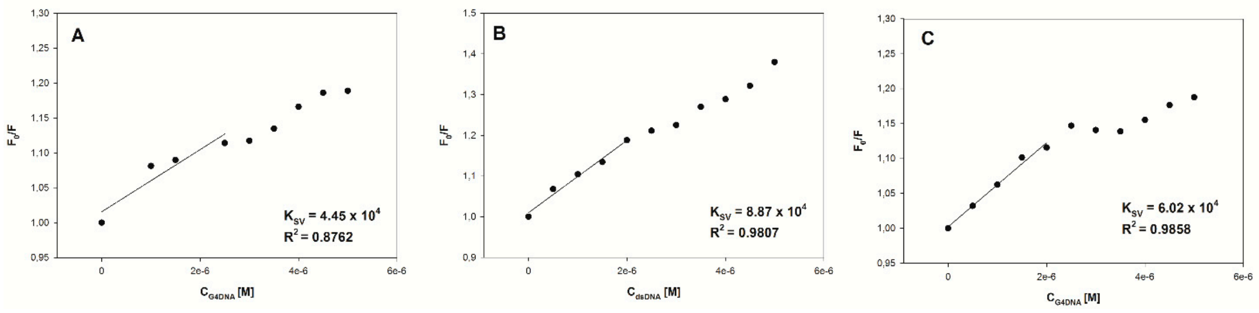

Figure S5. Stern-Volmer plots showing quenching of CdTe@COOH QDs fluorescence at 525 nm against DNA concentration. (A) G-quadruplex (CatG4 in 10 mM KCl), (B) ds DNA (CatG4/cCatG4 in 10 mM KCl), (C) ssDNA (CatG4 without KCl). 1 μM QDs in 10 mM Tris-HCl (pH = 8,0) was excited at 350 nm.

4. CD titration of GQ with QDs

This experiment was carried out by recording the circular dichroism spectra using the same spectral parameters as those for CD spectroscopic characterization of QD-DNA conjugates describen in Experimental. The G-quadruplex solution of CatG4 was prepared in 10 mM KCl and Tris-HCl buffer pH = 8.0 and prior to titration, the GQ solution was heated for 5 min at 90° C and then cooled in an ice bath for 15 min.

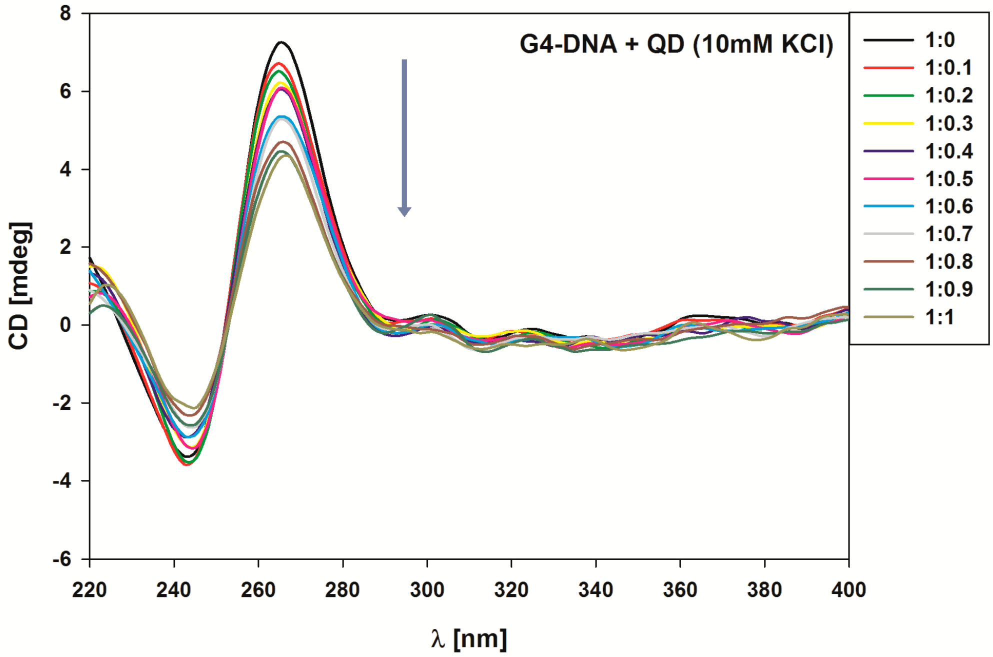

Figure S6. Circular dichroism spectra of 1 μM CatG4 G-quadruplex (GQ) solution in 10 mM KCl titrated with QDs.

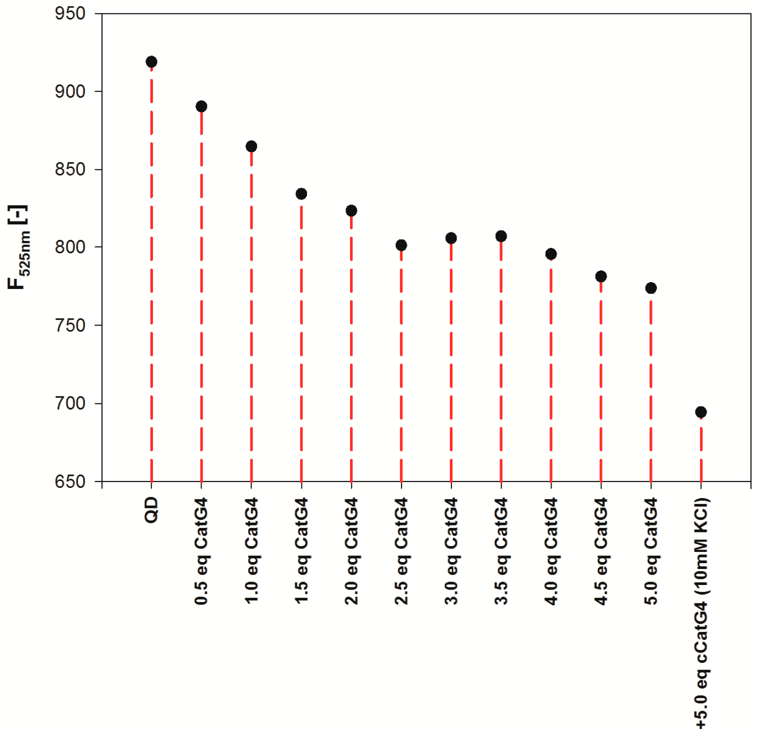

Figure S7. Dependence of the fluorescence intensity of 1 µM QDs upon sequential titration with CatG4 and its complementary strand cCatG4.

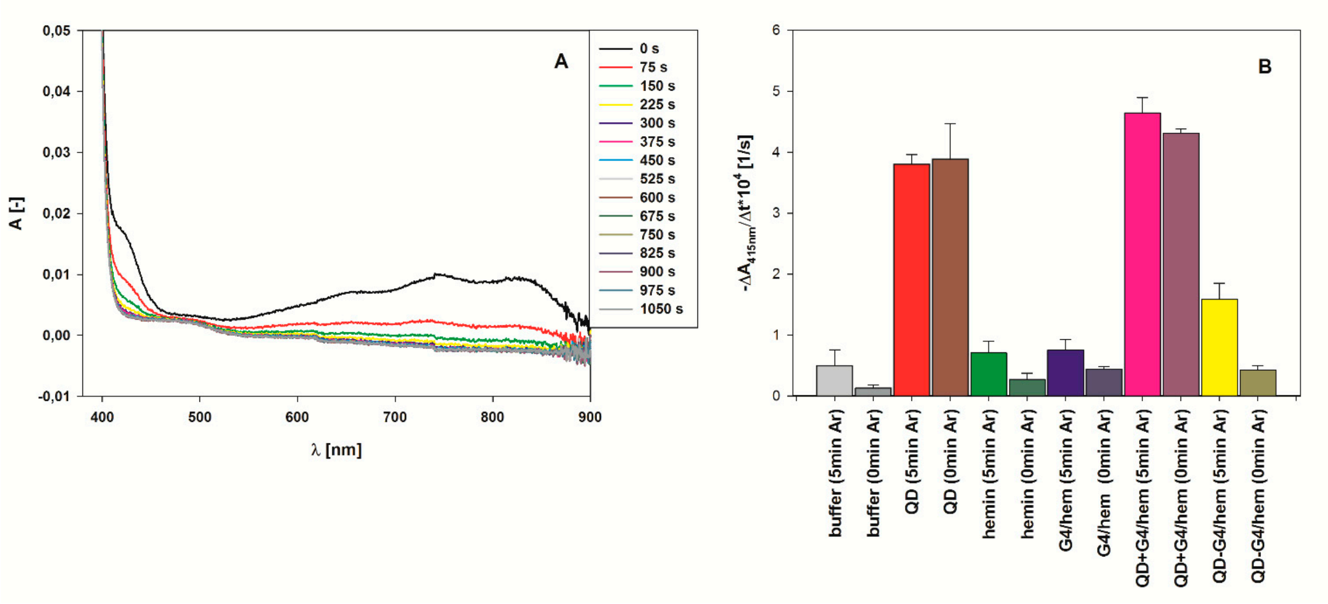

Figure S8. (A) Absorption spectra for QD-DNAzyme catalyzed oxidation of ABTS recorded at 0 - 1050 s, (B) Activity for studied systems with ABTS substrate in 10 mM Tris-HCl (pH=8,0); 10 mM KCl; 167 nM QD-DNA or QD / 500 nM DNA, 500 nM hemin; 1 mM ABTS, 0.1% DMSO. Activity was expressed by observing changes in absorbance at 415nm with time ($\Delta A_{415nm}/\Delta t$) and the irradiation time is 15 min using monochromatic light at 350nm.

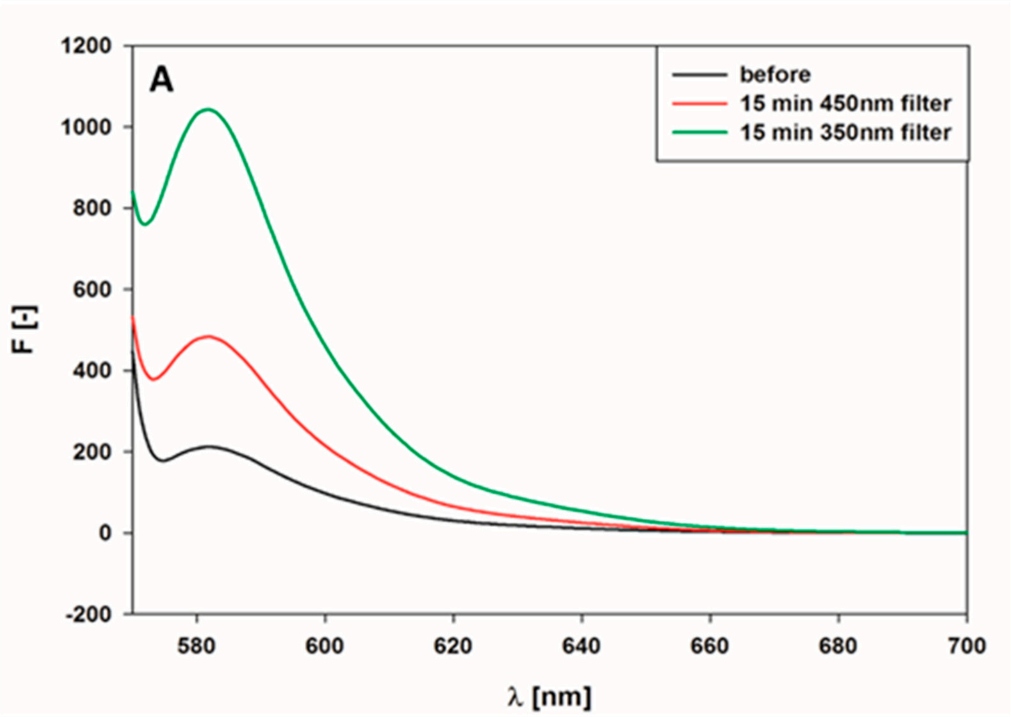

Figure S9. Fluorescence spectra of resorufin produced by QD-DNAzyme catalyzed oxidation of Amplex Red, recorded before (black line), and after 15 min irradiation using 450 nm filter (red line) or 350 nm filter (green line). Emission spectra with excitation at 560 nm (Conditions: 10 mM Tris-HCl (pH = 8,0); 10 mM KCl; 33 nM QD-DNA (100 nM G-quadruplex), 100 nM hemin; 5 μM Amplex Red, 1,1% DMSO).
